# ECMO and Impella Support Strategies as a Bridge to Surgical Repair of Post-Infarction Ventricular Septal Rupture

**DOI:** 10.3390/medicina58050611

**Published:** 2022-04-28

**Authors:** Garrett Coyan, Neesha Anand, Mahnoor Imran, Hernando Gomez, Raj Ramanan, Holt Murray, Saurabh Sanon, Pyongsoo Yoon, David Kaczorowski, Johannes Bonatti

**Affiliations:** 1 Department of Cardiothoracic Surgery, UPMC Heart and Vascular Institute, University of Pittsburgh, Pittsburgh, PA 15213, USA; coyangn@upmc.edu (G.C.); imranm3@upmc.edu (M.I.); yoonpd2@upmc.edu (P.Y.); kaczorowskidj2@upmc.edu (D.K.); 2 Department of Critical Care Medicine, University of Pittsburgh, Pittsburgh, PA 15213, USA; anandn@upmc.edu (N.A.); gomezh@upmc.edu (H.G.); ramananrr@upmc.edu (R.R.); murrhn@upmc.edu (H.M.); 3 Division of Cardiology, Department of Medicine, UPMC Heart and Vascular Institute, University of Pittsburgh, Pittsburgh, PA 15213, USA; sanons@upmc.edu

**Keywords:** ventricular septal rupture, ventricular septal defect, mechanical circulatory support, extracorporeal membrane oxygenation, Impella, post-infarction mechanical complication, cardiogenic shock

## Abstract

*Background and Objectives:* Post-infarct ventricular septal rupture (PIVSR) continues to have significant morbidity and mortality, despite decreased prevalence. Impella and venoarterial extracorporeal membranous oxygenation (VA-ECMO) have been proposed as strategies to correct hemodynamic derangements and bridge patients to delayed operative repair when success rates are higher. This review places VA-ECMO and Impella support strategies in the context of bridging patients to successful PIVSR repair, with an additional case report of successful bridging with the Impella device. *Materials and Methods*: We report a case of PIVSR repair utilizing 14 days of Impella support. We additionally conducted a systematic review of contemporary literature to describe the application of VA-ECMO and Impella devices in the pre-operative period prior to surgical PIVSR correction. Expert commentary on the advantages and disadvantages of each of these techniques is provided. *Results*: We identified 19 studies with 72 patients undergoing VA-ECMO as a bridge to PIVSR repair and 6 studies with 11 patients utilizing an Impella device as a bridge to PIVSR repair. Overall, outcomes in both groups were better than expected from patients who were historically managed with medicine and balloon pump therapy, however there was a significant heterogeneity between studies. Impella provided for excellent left ventricular unloading, but did result in some concerns for reversal of shunting. VA-ECMO resulted in improved end-organ perfusion, but carried increased risks of device-related complications and requirement for additional ventricular unloading. *Conclusions*: Patients presenting with PIVSR in cardiogenic shock requiring a MCS bridge to definitive surgical repair continue to pose a challenge to the multidisciplinary cardiovascular team as the diverse presentation and management issues require individualized care plans. Both VA-ECMO and the Impella family of devices play a role in the contemporary management of PIVSR and offer distinct advantages and disadvantages depending on the clinical scenario. The limited case numbers reported demonstrate feasibility, safety, and recommendations for optimal management.

## 1. Introduction

Despite the decrease in the incidence of post-infarction ventricular septal rupture (PIVSR) in the modern era due to early revascularization strategies, significant morbidity and mortality are common with this complication. A recent systematic review demonstrated that in more than 6000 patients the operative mortality for PIVSR repair of 34% has remained stable since the 1970s [1]. Proceeding with life-saving surgical repair can be challenging given that patients with PIVSR frequently present in overt cardiogenic shock, with early repair further being technically complicated by poor surgical tissue quality due to acute myocardial damage surrounding the PIVSR. Accordingly, patients may benefit from hemodynamic support to restore tissue perfusion and to prevent or reverse end-organ dysfunction, while providing enough time for the myocardium to scar and regain the consistency necessary for a successful surgical repair. Available support strategies include medical management based on the use of inotropes and vasopressors, and mechanical circulatory support (MCS) strategies.

To that end, a wide-ranging analysis of the Society of Thoracic Surgeons database demonstrated that approximately 65% of patients with PIVSR are treated with the insertion of an intra-aortic balloon pump (IABP) pre-operatively [2]. While the IABP remains the most common device inserted for support following PIVSR, the evidence for benefit in bridging remains mostly anecdotal, with no documented improvement in outcomes despite the large number of patients undergoing therapy. For this reason, more advanced MCS strategies have recently gained popularity in this sub-cohort of patients, but remain less well-established with only small reports describing efficacy [3].

Venoarterial extra-corporeal membrane oxygenation (VA-ECMO) and the Impella^®^ device (Abiomed, Inc., Danvers, MA, USA) are the two most common MCS strategies reported in the literature following IABP. Data on the pre-operative use of these strategies in PIVSR remain mostly limited to case reports and smaller case series. The aims of this review are: (i) to summarize the characteristics, advantages, and disadvantages of each of these modes of MCS; and (ii) to describe the reported frequency of the use of VA-ECMO and Impella for the hemodynamic stabilization of patients with PIVSR, the pre- and post-operative duration of support, and the rate of major post-operative outcomes and mortality, in the framework of an illustrative case.

## 2. Materials and Methods

### 2.1. Ethics Statement

Informed consent for reporting a de-identified case was obtained from the patient described in the case summary reported. No additional ethical considerations or approval were necessary for the systematic review portion of this work.

### 2.2. Case Report

A previously unreported case of a patient suffering from PIVSR successfully bridged to surgical repair with an Impella 5.5 device is presented to add to the extant literature.

### 2.3. Expert Overview and Commentary

A brief overview, along with advantages and disadvantages of each mode of MCS in the setting of PIVSR is provided by the multi-disciplinary authorship consisting of experts in cardiothoracic surgery, critical care medicine, and interventional cardiology.

### 2.4. Systematic Review of the Literature

A narrative-style systematic review of the literature was conducted utilizing the Medline and Embase databases. A meta-analysis was not planned due to the known heterogeneity in reporting and the isolated nature/overall low numbers of known cases reported.

#### 2.4.1. Search Strategy and Study Selection

Listed databases were searched utilizing the following search strategy: (Postinfarction ventricular septal defect OR postinfarction ventricular septal rupture OR ventricular septal defect OR ventricular septal rupture) AND (extracorporeal membrane oxygen OR ECMO OR Impella OR mechanical circulatory support OR ECLS). All case reports, case series, cohort studies, and clinical trials were considered appropriate primary literature for the purposes of this study examining PIVSR. While systematic reviews and secondary database studies were collated for background and review, they were not included in the primary reporting to eliminate duplicative reports in this small subset of patients. Patients were only included in the review if MCS was initiated prior to proceeding with surgical PIVSR. Abstracts identified were reviewed by three reviewers independently for inclusion into the study (GC, NA, JB) with subsequent full manuscript review and analysis.

#### 2.4.2. Data Extraction and Outcomes

Data to be extracted from each study was defined a priori. The variables obtained from each study included author, year of publication, number of patients, and duration of support before and after surgery. Clinically relevant outcomes extracted were post-operative ICU length of stay (LOS), hospital LOS, occurrence of any stroke, and all-cause mortality at any time after surgery.

#### 2.4.3. Statistical Analysis

Descriptive statistics culled from the literature were reproduced in the study’s tables for review. No comparisons were performed given the significant heterogeneity of the included study data.

## 3. Results

### 3.1. Case Report

A 64-year-old male with history of hypertension, diabetes, and hyperlipidemia presented in transfer from a referring hospital with a 5-day history of progressive chest pain and ST-segment elevation in inferior leads on 12-lead EKG. Prior to transfer, he underwent cardiac catheterization revealing 100% occlusion of the mid-right coronary artery with deployment of a drug-eluting stent and subsequent partial return of flow. He had respiratory decompensation requiring emergent endotracheal intubation, and was found to be SARS-CoV-2 positive with confirmed COVID-19 pneumonia. He further decompensated into cardiogenic shock and an IABP was placed with subsequent transfer to our institution; a posterior VSR was identified at that time. He was deemed high-risk for emergent surgical intervention at that time, and the decision was made to bridge him medically for a period of 3–4 weeks to allow time for recovery from his pneumonia and improvement in tissue quality for VSR repair. Due to the need for prolonged cardiac support, we elected to upgrade his support strategy to the Impella 5.5 that was placed surgically via the right axillary artery on hospital day 12. His course was complicated by a ventricular fibrillation cardiac arrest followed by recurrent ventricular tachycardia, despite adequate device position, and managed pharmacologically with amiodarone as well as synchronized cardioversion. Patient also developed a superimposed bacterial pneumonia requiring antibiotic therapy. On day 15 of hospitalization, patient developed acute blood loss anemia without escalating vasopressor requirements, serial non-contrast CT imaging demonstrating an iliopsoas muscle hematoma, stable retroperitoneal bleed, and right inguinal hematoma at the previous IABP site. Patient was managed conservatively with blood product transfusion and temporary discontinuation of anticoagulation and transition to a P2Y12 inhibitor infusion. He was eventually extubated, mobilized on device support, and optimized for definitive repair following clearance of his COVID-19 pneumonia on Impella day 13, hospital day 24. Of note, on Impella day 8 the device had to be exchanged due to a fracture in the driveline that occurred during patient maneuvering. Otherwise, he suffered no additional Impella-related adverse events.

By the day of surgery (day 29 following his infarct, hospital day 24), the patient had improved significantly from a respiratory and hemodynamic standpoint with recovery and stabilization of end-organ function. He underwent successful pericardial patch closure of a posterior 2 cm VSR via left ventriculotomy requiring no post-operative mechanical circulatory support. His post-operative left-ventricular ejection fraction was 35%, improved from 25% pre-operatively. He was extubated on post-operative day 1 and transferred out of the ICU on post-operative day 4. He progressed well during his recovery and was successfully discharged to a rehab facility on post-operative day 17.

### 3.2. Impella MCS Strategy in PIVSR

#### 3.2.1. Expert Overview and Commentary

##### Principles of Usage in PIVSR

An Impella is a percutaneously placed cardiac assist device that provides simultaneous hemodynamic support and myocardial protection that is available in several different models, tuned for various flows and access site considerations (Impella 2.5, CP, 5.0, and 5.5) [4,5]. It consists of a microaxial flow pump attached to a catheter which is positioned into the left ventricle retrograde across the aortic valve through the aorta via the femoral or axillary artery. Blood from the left ventricle is drawn into the inlet of the cannula and is delivered into the aortic root through the outlet in a continuous flow fashion with the ability to generate blood flows of up to 5.5 L/min (limited by the Impella device-type), thus increasing the total cardiac output. Unloading of the left ventricle reduces the left ventricular end diastolic volume and pressure, mechanical work, and myocardial wall tension, thus reducing myocardial oxygen demand. Increased aortic outflow pressure will reduce myocardial wall tension and also increase the myocardial oxygen supply by augmenting coronary flow. Direct unloading of the left ventricle can be particularly useful in PIVSR by decreasing left-to-right shunting; however, the patient’s native gas exchange must be adequate on this mode of MCS.

##### Potential Advantages

Direct unloading of the left ventricle with the Impella system has been postulated to be beneficial in the setting of PIVSR by decreasing left-to-right shunting. La Torre et al. implanted an Impella 5.0 in five patients with PIVSR and cardiogenic shock [6]. They noted a reduction in Qp (pulmonary flow): Qs (systemic flow) was reduced from 2.9 to 1.5 and SVO2 decreased from 88% to 76%, suggestive of reduced left-to-right shunting. Cardiac index increased from 1.9 to 3.1 L/min/m^2^ and central venous pressure as well as pulmonary capillary wedge pressure fell by 8 mmHg and 12 mmHg, respectively; this is a distinct advantage of devices aimed at left ventricular unloading. Resolution of the pulmonary edema after Impella insertion for PIVSR was also described by several groups, which is advantageous in the optimization of patients awaiting surgical PIVSR repair [6,7,8].

A major advantage with trans-axillary Impella implantation is the ability to allow for careful mobilization of the patient. This can be particularly useful during potential bridging strategies with the main goal of optimizing rehabilitative potential for delayed PIVSR surgical repair.

##### Potential Disadvantages

Despite being relatively easy to handle, Impella pump and device malfunctions have been reported. La Torre et al. described a pump exchange required due to high purge pressures suggestive of pump thrombosis [6]. In our case, pump exchange was required after a fracture in the driveline following patient mobilization. Lemaire et al. noted device malfunction in 5/47 patients (10.6%) undergoing Impella implantation for cardiogenic shock of various causes; high purge pressures were noted in 3/47 patients (6.4%) in the same series [9].

Even though the left-to-right shunt is reduced in the majority of cases, right-to-left shunting with subsequent desaturation can also occur. Maeda et al. described this in a case report of a VA-ECMO and Impella combination (ECPELLA) for bridging to surgical repair [10]. The right-to-left shunt with significant desaturation occurred during an ECMO weaning attempt. Full ECPELLA support was subsequently necessary until surgery. A similar case report was also described by Hiraoka et al. wherein an ECPELLA strategy caused a right-to-left shunt with desaturation [11]. This reversal of shunt flow may be exacerbated by right heart failure, making the assessment of right heart function imperative while on support. Operators should be especially vigilant about proper power levels and support status while on Impella in the setting of PIVSR for this reason.

Hemolysis is another complication of Impella support, often associated with the smaller Impella 2.5 pump when set at very high performance levels and subsequent high pump speeds. La Torre et al. reported a case of blood transfusion for possible hemolysis in a PIVSD patient supported with an Impella 5.0 [6]. Ibebuogu et al. described a case report of severe hemolysis in conjunction with a transitory ischemic attack prompting a change in the treatment plan from surgical to percutaneous closure secondary to high surgical risk [12]. Other risks include mechanical septal damage from the Impella pump and stroke.

Local infection of the Impella insertion site is a complication noted in one of the five patients in the series reported by Turin et al. [6]. This ultimately resulted in femoral artery rupture and death of the patient. For many of the aforementioned reasons, we prefer using the surgically implanted Impella 5.5, thus avoiding femoral artery complications and allowing for mobilization and ambulation of the patient.

#### 3.2.2. Review of Literature for Impella Bridging in PIVSR

There were 6 studies that included 11 patients meeting our inclusion criteria, utilizing an Impella device as the primary MCS strategy (Table 1) [6,7,8,13,14]. All included studies were case reports or small case series, with the largest reported series including five patients. Pre- and post-operative courses were highly variable between the groups with pre-operative duration of support ranging from 7–14 days, with 3 of the 11 patients reported on during the study period experiencing a mortality. Of note, no strokes were reported in this group of patients.

### 3.3. VA-ECMO as MCS Strategy in PIVSR

#### 3.3.1. Expert Overview and Commentary

##### Principles of Usage in PIVSR

VA-ECMO is a form of temporary mechanical circulatory support that provides cardiopulmonary support in patients with cardiac failure with or without concomitant respiratory failure [15]. The VA-ECMO circuit consists of a venous inflow cannula, pump, oxygenator and an arterial outflow cannula. Blood is drained from the venous system by a cannula that is inserted into the vena cava or right atrium either centrally, or peripherally most commonly via the femoral vein. Oxygenated blood is then returned to the arterial system through a cannula that is inserted into the ascending aorta if a central cannulation strategy is utilized, or peripherally into the subclavian or femoral artery. Physiologically, unloading of the right ventricle and pulmonary vascular system is achieved and systemic organ perfusion is improved. However, due to the retrograde flow of blood in the ECMO circuit into the aorta, the left ventricular afterload is increased; this can be problematic in PIVSR by increasing the shunt fraction [10]. In peripheral cannulation strategies, small antegrade distal perfusion catheters are often placed in the superficial femoral artery to maintain adequate distal limb perfusion [16].

##### Potential Advantages

An advantage of VA-ECMO support is that the method is more well-established at many institutions. Insertion is relatively rapid and straightforward in the hands of trained providers and can occur in a variety of clinical settings such as ICUs, ORs, cardiac catheterization labs, as well as in emergency rooms. Prompt improvement of hemodynamic and metabolic parameters are described in the papers we reviewed relating to PIVSR. Takaki et al. reported a fall in pulmonary artery pressure from 50/19 mmHg to 18/10 mmHg within a day, as well as resolution of pulmonary edema and liver congestion within 5 days of VA-ECMO implantation [17].

VA-ECMO is an excellent option for reliably restoring end-organ perfusion in patients with PIVSR. Muller Moran et al. noted improvement of a patient’s metabolic status and normalization of arterial lactate concentration within 24 h of ECMO support [18]. A dramatic fall in lactate levels from admission to pre-operatively was also described by Doi et al. [19].

Concerning one year survival after PIVSR, Rob et al. noted 0% survival in patients in shock without VA-ECMO support [20]. With VA-ECMO support, survival rose to 30% in this small series. Patients without shock survived one year at a rate of 50%. In a larger recent report, Malik et al. reported an operative mortality of 11% and a 1-year survival of 66% in patients being bridged to surgical repair with MCS; of the 27 patients included in their study, 21 were supported with VA-ECMO [21].

##### Potential Disadvantages

The most prevalent complication on VA-ECMO support is bleeding. Rob et al. observed major bleeding in 71.4% of patients and bleeding was fatal in 42.9% of patients with PIVSR supported with ECMO [20]. Bleeding occurred both in the thoracic operative field and in the groin after cannulation. Artemiou et al., in a small series of three patients, noted bleeding with the necessity of surgical revision in all cases [22]. They suggested targeting an activated clotting time (ACT) of 120 to 180 s and to even run the VA-ECMO circuits without anticoagulation for a few days, if necessary. Rohn et al. followed a target ACT of 140 to 150 s [23]. Bleeding can be aggravated because the many patients with PIVSR may have undergone acute PCI and are on dual antiplatelet therapy [19].

Another important complication is leg ischemia after femoral arterial cannulation. Kwon et al. described leg ischemia four days after ECMO implantation, prompting the decision for surgical repair of PIVSR at that time point [24]. Rob et al. reported this complication in PIVSR patients with a frequency of 14.3% [20]. Use of a femoral arterial return cannula smaller than 19 F was suggested by Artemiou et al. and prophylactic installation of a wire-reinforced distal perfusion cannula is common practice at many institutions [22]. Primary axillary artery cannulation is another option to prevent this complication [25]. Rohn et al. describe use of a femoral artery side graft for adequate perfusion of the leg [23]. Vascular injury such as perforation and dissection during insertion is also a concern specifically in patients with advanced coronary artery disease and generalized vasculopathy.

VA-ECMO is additionally contraindicated in patients with more than mild aortic valve regurgitation. With a proper closing aortic valve, however, no left ventricular unloading may be necessary, as described by Doi et al. in his series of six PIVSR patients [19]. Nevertheless, close monitoring of left ventricular distension is indicated in these patients as it may exacerbate shunting and lead to increased pulmonary edema and lung injury. Left ventricular venting via a direct catheter or placement of Impella (ECPELLA) may be indicated in these cases and should be completed in a timely fashion [26]. Neragi-Miandoab et al. reported that, despite adequate VA-ECMO flows, the pulmonary overflow could not be controlled and their patient was therefore taken to the operating room for definitive repair [25]. Vigilance also must be applied to prevent overflow of the Impella portion of ECPELLA which can lead to shunt reversal [11].

Installation of advanced mechanical support devices carry the risk of contamination with the potential for overt infection and sepsis. Rob, in his series of PIVSR patients receiving VA-ECMO pre-operatively, reports a 43% rate of pneumonia and a corresponding 57% rate of sepsis [20]. One reported approach to prevent pneumonia is to extubate the patient on VA-ECMO in patients with successful surgical treatment of PIVSR [27]. Early mobilization can also be utilized, but this can be more difficult depending on the VA-ECMO cannulation strategy.

#### 3.3.2. Review of Literature for VA-ECMO Bridging for PIVSR

There were 19 studies that included 72 patients meeting our inclusion criteria for outcomes reporting utilizing VA-ECMO device as the primary MCS strategy (Table 1) [18,19,20,21,22,24,25,27,28,29,30,31,32,33,34]. Most included studies were case reports or small clinical series, with the largest cohort study reporting 21 patients. Pre- and post-operative courses were highly variable between the groups with 19 of the 72 patients reported on experiencing mortality with a median pre-operative MCS support duration ranging from 1.5 to 16 days among the reported studies. This wide range of clinical events is due to the differences in strategies employed in these studies, as some had specific goals to extend the bridging period to allow for PIVSR maturation, while others simply sought to bridge a critically ill patient in cardiogenic shock to emergent surgical repair. While the largest range of experiences with VA-ECMO in mechanically complicated myocardial infarctions comes from the Extracorporeal Life Support Organization (ELSO) database, the only information provided about pre-operative VA-ECMO specifically was that eight patients underwent VA-ECMO placement and subsequently had surgical correction while on support; there was a high drop-out between the number of patients initially put on VA-ECMO and those making it to surgical correction, indicating high mortality pre-operatively at least in this cohort [35].

## 4. Discussion

### 4.1. Interpretation of Current Literature

Though its prevalence has decreased, PIVSR continues to have significant morbidity and mortality that has not substantially changed over the last decades [36,37]. The management of PIVSR therefore includes resuscitation from cardiogenic shock and stabilization until surgical closure is performed. Longer durations from acute myocardial infarction to PIVSR and from PIVSR to surgery have been associated with lower mortality [3]. This improved survival in those with a longer interval before repair may be due to evolution of the infarct and scar tissue formation prior to intervention. The optimal timing of PIVSR repair, however, remains unclear. A PIVSR can occur in the initial 24 h post-myocardial infarction (MI), but generally develops 3–8 days later. As per the Society of Thoracic Surgeons National Database, operative mortality was 54.1% if repair was ≤7 days from MI and 18.4% if >7 days from MI [2]. Animal studies have observed microscopic collagen detection by day 7 post-infarct and by day 28, necrotic myocytes are predominantly replaced by fibrotic tissue. Proteolytic enzyme activity peaks 3–4 days after infarct, making myocardial tissue highly friable. The remodeling phase, wherein infarct scar formation occurs, can last up to months in humans [38]. Therefore, early intervention may lead to a post-op residual shunt due to a weak myocardium ineffectively held by sutures and the optimal time to surgical intervention may be closer to 4 weeks following development of PIVSR. It is this role that MCS strategies such as VA-ECMO and Impella could successfully occupy in contemporary treatment, according to our review [14,33,39,40].

A bias may also exist, given that patients that are generally intervened upon sooner are increasingly hemodynamically unstable with significant end-organ hypoperfusion, which may also contribute to the increased mortality noted with early intervention. PIVSR can present as hemodynamic stability to florid cardiopulmonary collapse, depending on the size of the defect, presence of RV infarction, ongoing RV ischemia, and/or stunning of the RV from volume overload. These patients can also deteriorate rapidly, thus requiring supplementary hemodynamic support dependent on the pathophysiology of dysfunction. The goal then is to reduce left-to-right shunt with afterload reducing agents and to bridge to surgical repair with MCS by providing hemodynamic stability and improving end-organ perfusion. While the majority of patients are currently supported with IABP, there is no strong evidence suggesting that it is an effective strategy [1]. The use of other modes of MCS such as VA-ECMO and Impella are potentially useful adjuncts in the stabilization efforts in PIVSR. The choice between VA-ECMO and Impella should be individualized based on patient presentation, center availability and expertise, and desired timing of surgical intervention [41]. While no head-to-head comparisons exist, simulation studies comparing MCS strategies suggest that the Impella may provide optimal ventricular unloading and shunt reduction. However, it should be noted that no respiratory support is provided with Impella, in contrast with ECMO. More research is warranted in this area.

Pre-operative MCS may improve hemodynamic and metabolic status and allow for delayed surgical intervention, but it can come at the cost of a high rate of complications [16,42]. Vascular access site-related complications can range significantly in severity from infection, superficial hematomas, and retroperitoneal bleeding managed conservatively to hemorrhagic or septic shock. Though our patient was found to have a stable small retroperitoneal bleed and inguinal hematoma at the site of the previously placed IABP, we were able to effectively manage conservatively without additional complications. Studies have also demonstrated that older age and severe comorbidities can be associated with a higher risk of vascular complications [43]. Therefore, bridging the patient out to at least 3–4 weeks post-VSR to obtain surgical tissue quality must be weighed against the risk of MCS-related complications. Interestingly, though we waited until hospital day 24 post-MI to intervene surgically on our patient, intra-operative findings demonstrated some visible scar formation, but the infarcted tissue was still relatively soft. Therefore, additional investigation into the optimal duration of bridge to VSR repair is necessary.

### 4.2. Limitations

This is a systematic review of small observational studies, precluding any possibility to compare time of support or outcomes between mechanical support strategies. In addition, the number of published studies of advanced mechanical support in postinfarction VSD is very low, suggesting an important lack of evidence in this space. Our review is prone to reporting bias as published studies tend to report successfully bridged cases in PIVSR. It is unclear how many additional patients have been supported with these strategies who have succumbed to multi-organ failure or have not reached the point of benefitting from surgical correction [3,19]. Finally, the reported studies that were presented are highly heterogeneous precluding the pooling of data and meta-analysis; this has been a consistent limitation in this field [3].

## 5. Conclusions

Both VA-ECMO and the Impella family of devices play a role in the contemporary management of PIVSR and offer distinct advantages and disadvantages, depending on the clinical scenario. The limited case numbers reported demonstrated feasibility, safety, and recommendations for optimal management. Utilization of these devices may allow for a period of hemodynamic stabilization, end-organ resuscitation, and an extended time of stability to delay definitive surgical closure of PIVSR to a time when tissue handling will be more technically facile. However, this must be balanced with the possibilities of unique MCS complications; current literature is not robust enough to elucidate the optimal timing for surgical repair in this regard. Additional prospective multi-institution studies would be welcome to evaluate these MCS options in a more robust and translational manner.

## Figures and Tables

**Table 1:** Primary Data Obtained Via Systematic Review of Impella and VA-ECMO Bridge to PIVSR Repair.

	First Author	Year Published	Number of Patients	Pre-Operative MCS Duration (Median Days)	Post-Op MCS Duration (Days)	Post-Op ICU Stay (Median Days)	Post-Operative Length of Stay (Median Days)	Stroke	Mortality
**Impella**									
	Patane	2010	1	14	NA	NA	NA	NA	0 (0%)
	La Torre	2011	5	14.5	NA	11.8	18.6	NA	3 (60%)
	Ancona	2017	1	7	NA	NA	28	NA	0 (0%)
	Iida	2019	1	15	3	5	15	0	0 (0%)
	Via	2020	2	7.5	0	7.5	20	0	0 (0%)
	Coyan	2022	1	13	0	4	17	0	0 (0%)
**VA-ECMO**									
	Niragi-Miandoab	2013	1	6	5	NA	NA	0	0 (0%)
	Hobbs	2015	3	4	0	NA	23	1	1 (33.3%)
	Huang	2015	6	NA	NA	NA	NA	NA	2 (33.3%)
	Kwon	2016	1	4	0	3	14	0	0 (0%)
	Mc Laughlin	2016	3	5.5	10.5	NA	NA	0	0 (0%)
	Park	2017	1	9	0	NA	18	0	0 (0%)
	Rob	2017	5	12	NA	NA	NA	NA	2 (40%)
	Rozado	2017	1	5	0	3	10	0	0 (0%)
	Chen	2018	1	8	0	NA	10	0	0 (0%)
	Muller- Moran	2018	1	9	6	38	86	0	0 (0%)
	Ram	2019	2	6	0.5	NA	30.5	0	0 (0%)
	Artemiou	2020	3	13	5.5	NA	NA	NA	1 (33.3%)
	Takaki	2020	1	7	0	NA	28	0	0 (0%)
	Artemiou	2020	3	12	5.5	NA	NA	0	1 (33.3%)
	Ariza-Sole	2020	7	5	4	NA	NA	0	2 (28.5%)
	Morimura	2020	5	1.5	0	5	23	1	1 (20%)
	Gambro	2021	1	16	4	NA	NA	NA	1 (100%)
	Malik	2021	21	NA	NA	NA	NA	2	7 (33.3%)
	Doi	2022	6	7	6.1	22.7	37	0	1 (14.3%)

## Data Availability

No data generated beyond the review completed within the manuscript.
